# A ranking of diffusion MRI compartment models with in vivo human brain data

**DOI:** 10.1002/mrm.25080

**Published:** 2013-12-17

**Authors:** Uran Ferizi, Torben Schneider, Eleftheria Panagiotaki, Gemma Nedjati-Gilani, Hui Zhang, Claudia A M Wheeler-Kingshott, Daniel C Alexander

**Affiliations:** 1 Department of Computer Science and Centre for Medical Image Computing, University College LondonLondon, UK; 2 NMR Research Unit, Department of Neuroinflammation, Institute of Neurology, University College LondonLondon, UK

**Keywords:** diffusion magnetic resonance imaging, brain imaging, white matter, microstructure imaging

## Abstract

**Purpose:**

Diffusion magnetic resonance imaging (MRI) microstructure imaging provides a unique noninvasive probe into tissue microstructure. The technique relies on biophysically motivated mathematical models, relating microscopic tissue features to the magnetic resonance (MR) signal. This work aims to determine which compartment models of diffusion MRI are best at describing measurements from in vivo human brain white matter.

**Methods:**

Recent work shows that three compartment models, designed to capture intra-axonal, extracellular, and isotropically restricted diffusion, best explain multi-*b*-value data sets from fixed rat corpus callosum. We extend this investigation to in vivo by using a live human subject on a clinical scanner. The analysis compares models of one, two, and three compartments and ranks their ability to explain the measured data. We enhance the original methodology to further evaluate the stability of the ranking.

**Results:**

As with fixed tissue, three compartment models explain the data best. However, a clearer hierarchical structure and simpler models emerge. We also find that splitting the scanning into shorter sessions has little effect on the ranking of models, and that the results are broadly reproducible across sessions.

**Conclusion:**

Three compartments are required to explain diffusion MR measurements from in vivo corpus callosum, which informs the choice of model for microstructure imaging applications in the brain. Magn Reson Med 72:1785–1792, 2014. © 2013 The authors. Magnetic Resonance in Medicine Published by Wiley Periodicals, Inc. on behalf of International Society of Medicine in Resonance.

## INTRODUCTION

Diffusion MRI measures water diffusion in biological tissue, which can be used to probe the microstructure. In brain imaging, the standard model for water dispersion in tissue is the diffusion tensor (DT) 1, which assumes a trivariate Gaussian dispersion pattern. This assumption of Gaussian diffusion oversimplifies the diffusive behavior of water in complex media, and is known experimentally to break down for relatively large *b*-values. DT derived indices, such as mean diffusivity or fractional anisotropy, can correlate with major tissue damage, e.g., in ischaemic brain injury 2, but lack sensitivity and specificity to subtle pathological changes.

Multicompartment models enable the estimation of more specific indices, such as axon diameter, density, orientation, and permeability, and so potentially give much greater insight into tissue architecture and sensitivity to pathology. Stanisz et al. 3 pioneered the representation of separate compartmental diffusive processes in nervous tissue with a three compartment model consisting of: ellipsoidally restricted intra-axonal water, anisotropically hindered extracellular water, and isotropically restricted glial cell water. This was followed by the Ball-Stick model 4, which is intended as the simplest model that separates intra- and extra-axonal water signals. Later, the composite hindered and restricted model of diffusion (CHARMED) 5 adds complexity by using cylinders with radii that follow a Gamma distribution and anisotropic hindered compartment for intra- and extra-cellular water, respectively. The AxCaliber technique 6,7 uses CHARMED to estimate axon diameter distribution and density. The ActiveAx technique 8,9 uses single diameter cylindrical restriction for intra-axonal water (simplifying the corresponding compartment of CHARMED), anisotropically hindered extracellular water, and a “Dot” compartment (simplifying Stanisz's isotropically restricted glial cell compartment). Later versions of ActiveAx 10 also accommodate dispersed orientations of the cylinders.

To identify which model compartments are essential to explain the data and parameters that are potentially estimable from a particular experiment, Panagiotaki et al. 11 built a taxonomy of one, two, and three compartment models, including the models from 3–6,8. They compared the models to each other using the Bayesian Information Criterion (BIC), ranking them in order of how well they explain data acquired from the fixed white matter of rat corpus callosum (CC). The study concluded that three compartment models with non-zero axon diameter, an anisotropic extracellular compartment, and an isotropic restriction model perform best. However, these results do not directly inform in vivo human imaging experiments because: (a) the tissue sample is from a small animal; (b) the tissue is fixed, which affects water diffusion significantly 12 and, therefore, different models may perform better; and (c) the experiment used an animal scanner that can achieve higher gradient strengths than human imaging systems.

This note extends the investigation in 11 to in vivo by performing a similar experiment using a live human subject on a clinical scanner. Specifically, we use a rich, massively multishell high angular resolution diffusion imaging protocol, to probe a wide range of gradient orientations, diffusion times, gradient pulse times, and gradient magnitudes. We extend the analysis in 11 by using bootstrapping and prediction of unseen data, to provide additional insight into the stability and accuracy of the model ranking.

## METHODS

This section describes the acquisition protocol for our data and outlines the preprocessing we do to obtain a set of measurements for fitting the models. It then details the fitting procedure, the technique we use for comparing the models and, lastly, the bootstrap procedure.

### Data Acquisition and Preprocessing

The central aim in this acquisition is to cover as large a portion of the measurement space as possible, while retaining a usable signal-to-noise level. The full protocol has 32 shells of 45-directions each. The set of directions in each shell is a unique random rotation (to enhance overall angular resolution) of the 45-direction Camino 13 point set. Each shell has a unique combination of gradient strength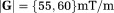, pulse width *δ* = {6, 10, 15, 22} ms, pulse duration Δ = {30, 50, 70, 90} ms, and has three interwoven *b* = 0 acquisitions. The *b*-values thus range from 218 to 10,308 s/mm^2^, with effective diffusion time (Δ−*δ*/3) in the range 28–82 ms. We use a Pulsed-Gradient Spin-Echo [24] sequence on a 3T Philips scanner, with cardiac gating and 4 s repetition time. The echo time varies between shells depending on the values of *δ* and Δ and is kept to a minimum to maximize signal. There are nine 4 mm thick sagittal slices, acquired with a reduced field-of-view using a ZOnally-magnified Oblique Multislice (ZOOM) Echo Planar Imaging (EPI) technique with outer volume supression 14. The field-of-view is centered on the midsagittal slice of the CC, where we assume that coherently oriented CC fibers are perpendicular to the image plane. The image size is 64 × 64 and the in-plane resolution is 2 × 2 mm^2^.

The study was approved by the local ethics committee, and written informed consent was obtained from the participant. We acquire the full protocol in a 31-year-old healthy subject in two different ways. The first full data set is acquired in two separate nonstop sessions, each lasting about 4 h 30 min; we refer to this as the 2 × 4 h data set. (We used dynamic stabilization facility provided by the scanner, which is designed for long scans to correct for field drifts during the image acquisition. We visually inspected the images and did not observe any obvious shifts from gradient heating.) We then repeat the protocol in eight sessions, each lasting 1 h 15 min; we call this the 8 × 1 h data set.

### Voxel Selection

We carefully manually registered the sagittal slices, where the in-plane correction was usually of order 1–3 voxels, and the quality of registration was confirmed visually for each individual image. All nondiffusion-weighted images are registered to the first unweighted image of the *b* = 1202 s/mm^2^ shell; the corresponding transformations are then applied to the fifteen diffusion weighted images that follow each *b* = 0 acquisition, as ordered in the scanning protocol. In this *b* = 1202s/mm^2^ reference shell, we manually segment the subject's CC, and then fit the DT to select a set of voxels with coherently oriented fibers. In particular, all voxels with FA > 0.6 and principal eigenvector within *η* = 2° of the assumed fiber direction (perpendicular to the image plane, i.e., left-right in the brain) are retained. In the 2 × 4 h data set, there are 24 voxels that satisfy the imposed criteria, all belonging to the two slices closest to the midsagittal plane. A similar procedure with *η* = 5° leaves 66 voxels and *η* = 10° which leaves 99 voxels. In the 8 × 1 h data set, 60, 101, and 166 voxels remain, respectively, sampling the CC rather more evenly; the same thresholding procedure leads to a slightly different set of voxels because of noise, misalignments, etc. To account for different echo time affecting different shells, the signal in each shell is normalised by the average of the three unweighted measurements (*b* = 0) with the same echo time.

**Figure 1:**
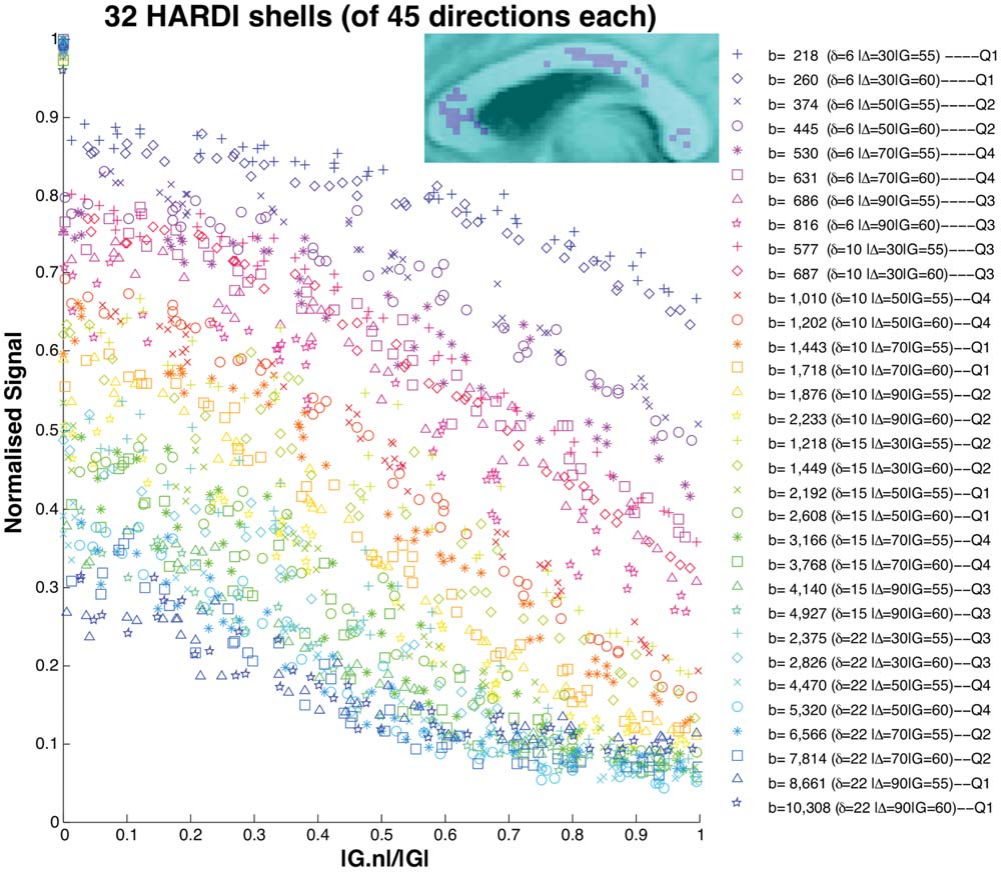
The acquired signal for the 2 × 4 h 2° data set. The legend gives *b*-value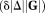 in units of s/mm^2^(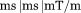); Q1–Q4 on the right define the four quarters of the full protocol used in the cross-validation (see “Cross-validation” section). The insert picture is of the CC and the selected voxels. G is the applied gradient vector and n is the fiber direction; the *x*-axis gives the absolute value of the cosine of the angle between the applied gradient and fiber direction: to the left, the gradient is perpendicular to the fibers; to the right, parallel.

As in 11, we create a single data set for each *η* = 2°, 5°, and 10° by averaging over the voxels selected above. Figure 1 shows the signal from the 2 × 4 h data set with *η* = 2° and confirms the rich coverage of the measurement space the protocol provides. The data sets contain 1356 = 32*(3 + 45) measurements each.

### Model Description

**Figure 2:**
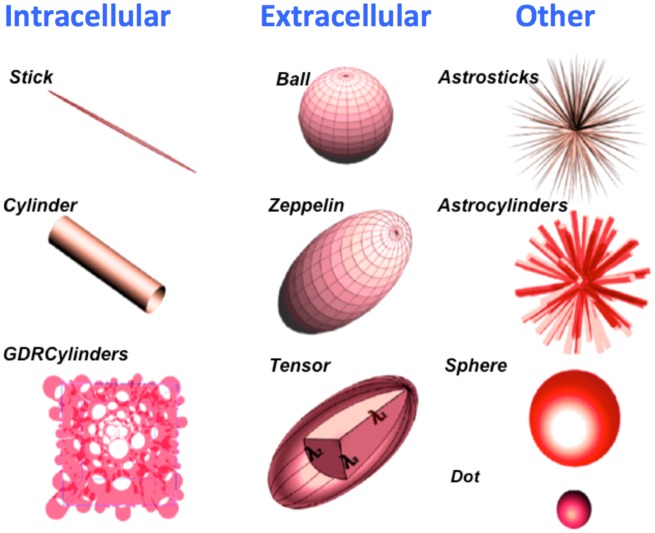
Model compartments, as designed to capture intracellular diffusion (left), extracellular diffusion (middle), and diffusion in other media (right).

Using the taxonomy of 11, as described in Figure 2, the extracellular compartment, “hindered” in 3D, can be: a Tensor (full DT), a Zeppelin (cylindrically symmetric DT), or a Ball (isotropic DT). The intracellular compartment, “restricted” in 2D but free in the other direction (anisotropic restriction), can be: a Stick (a spatially oriented line), a Cylinder (a stick with non-zero radius), or GDR-cylinders or GDRcylinders (Cylinders with a Gamma distribution of radii; the distribution is characterized by shape parameter *κ* and scale parameter *θ*, where *κθ* is the distribution's mean, and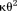 gives its variance). A special case is the Bizeppelin, which combines two cylindrically symmetric tensors (a 3D biexponential model). In three compartment models, the isotropically restricted third compartment can be: a Sphere (where diffusion is restricted to within a sphere of non-zero radius), a Dot (zero radius Sphere), Astrosticks (Sticks isotropically distributed in 3D), or Astrocylinders (Cylinders isotropically distributed in 3D).

### Model Fitting

We fit the models described above to each data set via the open source Camino toolkit 13. As in 11, the fitting uses the Levenberg–Marquardt algorithm with a perturbed starting point initialized from less complex models and same parameter constraints. Each model is fitted 250 times, and the final parameters are those that produce the minimum objective function.

The objective function, the Least Squares Error1
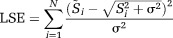
uses an offset-Gaussian noise model, where *N* is the number of measurements,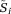 is the *i*th measured signal, *S_i_* its prediction from the model; *σ* = 0.05 is the noise standard deviation, which we estimate a priori from the *b* = 0 signals. This objective function accounts for the Rician noise inherent in the MRI data 15,16 and is simpler and more numerically stable than a full Rician log-likelihood objective function.

### Model Selection

The fitting metric LSE does not account for model complexity (as the most complex model would always win), so it cannot be used to indicate performance. The model selection mechanism we use is the Bayesian Information Criterion 172
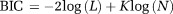
where *L* is the likelihood of model parameters given the data and *K* is the number of free parameters. The BIC accounts for complexity by adding a penalty term to the chi-squared measure (adapted for Rician noise). We evaluate the BIC for each fitted model and then rank all models from lowest BIC (best) to highest (worst).

### Bootstrapping

We use classical bootstrap 18 to analyze the stability of the BIC ranking. Each bootstrap data set comes from a random selection in each shell of the same number of data points, with replacement. For each 2 × 4 h and 8 × 1 h data set, we construct 100 bootstrap data sets; we then obtain 100 BIC rankings after fitting the models to each data set 50 times and picking the best parameter estimates. We construct positional variance diagrams, which give the number of times out of 100 that each model appears in each position in the ranking.

### Cross-Validation: Predicting Unseen Data

Cross-validation provides a complementary model selection to confirm the findings from the BIC. We use four-fold cross-validation and divide the data set into four quarters as shown in Figure 1. Each quarter is constructed by dividing all the shells of each *δ* into two groups of low Δ (30 and 50 ms) and high Δ (70 and 90 ms). Then, we randomly assign one from each group to each quarter; shells with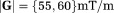 go together.

The cross-validation then proceeds as follows: we fit the models to data from three quarters of the protocol's data; then we synthesise data for the remaining quarter of the protocol, using the best fit parameter estimates, and evaluate the LSE in Eq. 1 compared to measured signal.

## RESULTS

**Figure 3:**
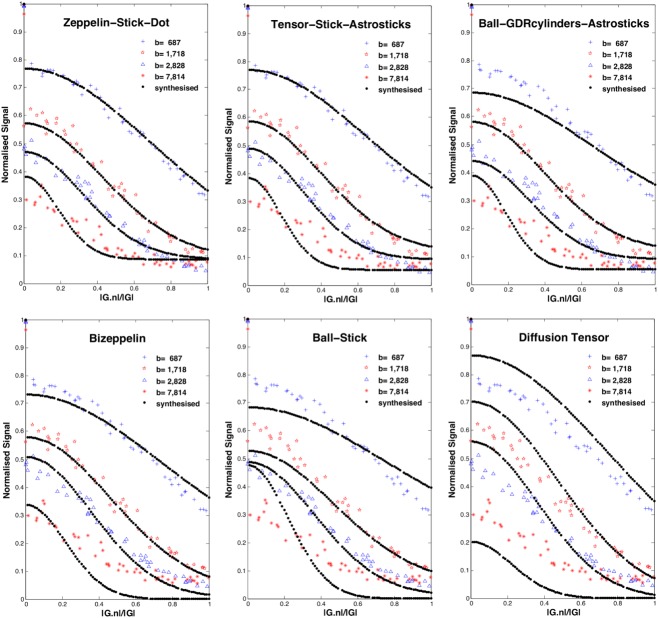
Synthesised signal, shown as dotted line, using the best parameter estimates from six representative models. This is superimposed on raw data, marked with red/blue colors; for clarity, we only show four shells across the sampled range of *b*-values. [Color figure can be viewed in the online issue, which is available at wileyonlinelibrary.com.]

Table1 shows some of the models ranked top-to-bottom by their BIC with selected parameter estimates for the *η* = 2° 2 × 4 h data set (Supporting Information Tables S1 and S2 gives the complete model rankings and parameter estimates). Several distinct groups of models emerge: (i) three compartment models with anisotropic extracellular compartment (Zeppelin/Tensor) and Dot/Sphere third compartment, which produce the best fit (and lowest BIC); (ii) three compartment models with anisotropic extracellular compartment and Astrostick/Astrocylinder third compartment, which are consistently worse than Dot/Sphere equivalents, but better than all other models; and (iii) three compartment models with isotropic extracellular compartment and all two compartment models. The DT comes below group (iii). The performance boundaries between the groups are very clear. Within the groups, as expected, the LSE consistently reduces as the complexity increases, and the BIC ranking rewards simpler compartments, but there is little to choose between the models in group (i). Figure 3 illustrates the fit of some of the models to the data. The models are fitted to a total of 32 shells, but we select only four to illustrate visually where the models over/under-estimate the signal. While the fitting is not perfect even for the best model of the ranking, the figure reflects clearly the model ranking in the signal prediction.

**Table 1:** Various Model Parameters from Data Sets, 2 × 4 h and 8 × 1 h, with Different Angular Thresholds of 2^o^, 5^o^, and 10^o^ (see Subsection Voxel Selection)

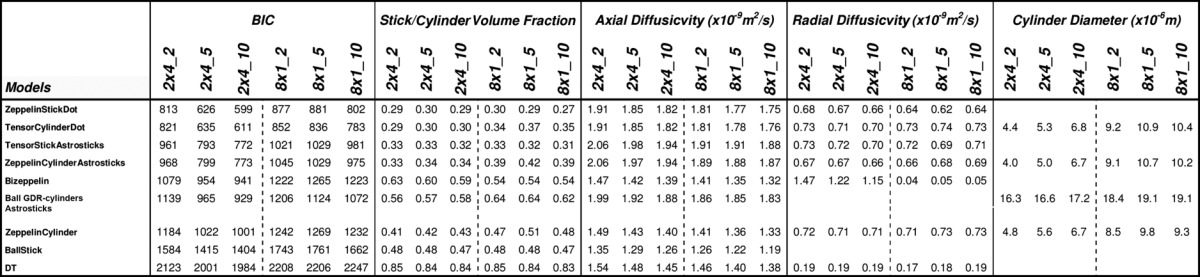

**Figure 4:**
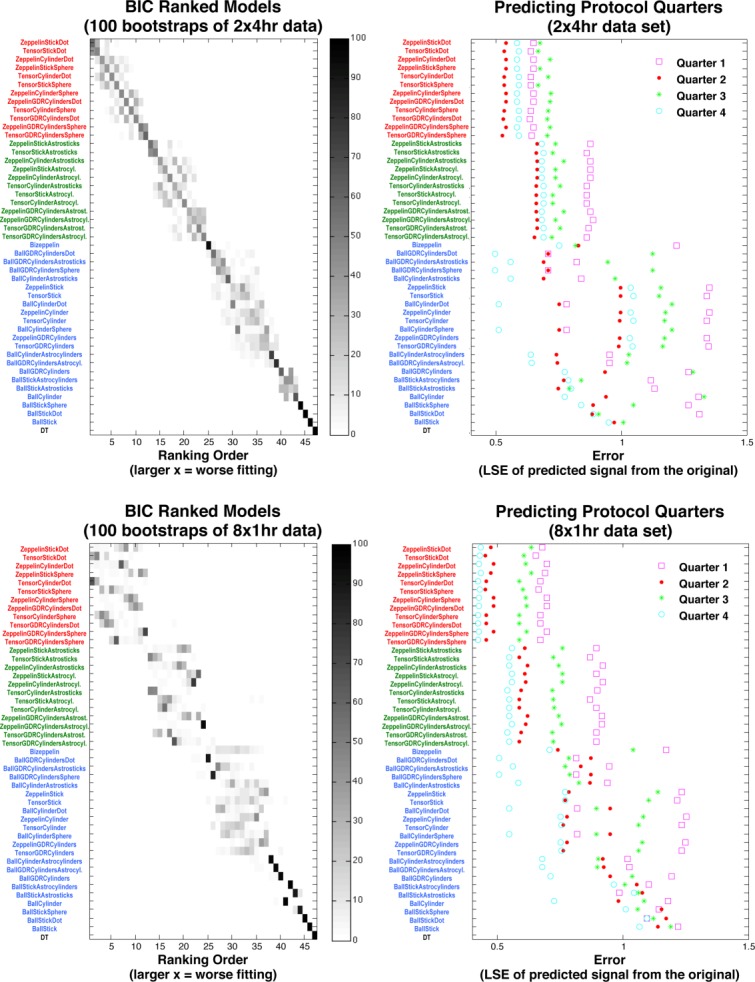
Left: Positional variance diagrams over 100 bootstraps from the 2 × 4 h (top-left matrix) and 8 × 1 h (bottom-left) 2° data sets. The frequency of *x*-axis ranking is given by the shade of gray; e.g., the Tensor comes out last in all 100 Bootstrap samples of 8 × 1 h. Right: The accuracy of predicting unseen quarters of the protocol using parameters fitted to data from the remaining three quarters. Each point is the LSE between the synthesized and measured signal. The top-bottom BIC ranking of models shows the color-coded group clusters, starting with three compartment models with anisotropic extracellular compartment and Dot/Sphere third compartment (in red), followed by those with Astrostick/Astrocylinder third compartment (green); next, three compartment models with isotropic extracellular compartment and all two compartment models (blue) go before DT. [Color figure can be viewed in the online issue, which is available at wileyonlinelibrary.com.]

Figure 4 shows the positional variance diagrams of model ranking over 100 bootstrap samples from both the 2 × 4 h and 8 × 1 h *η* = 2° data sets. The group structure of the ranking is very consistent over the bootstraps, although we see some variance of model positions within the groups; the ranking is also consistent between the 2 × 4 h and 8 × 1 h data sets, though some difference is expected, arising from minor imperfections in the registration of images in such large data sets. The group structure is also similar for the *η* = 5° and 10° data sets (results not shown). Differences in the number of voxels averaged in these datasets have little effect on the rankings. The plots on the right-hand side of Figure 4 show results from cross-validation. The same group structure emerges with on average group (i) performing best, followed by group (ii), and more erratic performance in group (iii). Little distinguishes models within group (i) or group (ii).

The parameter estimates in Table1 (and other Supporting Information Tables S1 and S2) show strong consistency within the groups but more variation between groups. In group (i), the intracellular volume fraction is unexpectedly low and about half of the extracellular volume fraction. One possible explanation is a significant free water contribution 6,7 which we do not model explicitly, and so gets absorbed in the extracellular component. Significant within-voxel fiber dispersion 10,19,20 could also cause this observation, as group (ii), which to some extent model fiber dispersion, show higher intracellular and third compartment volume fractions.

In hindered compartments, the axial diffusivities in groups (i) and (ii) are consistently around 2 × 10^−9^ m^2^/s, and the radial diffusivities are around 0.7 × 10^−9^ m^2^/s, in agreement with previous reports 21. The two radial diffusivities of Tensor models are close, making Tensor and Zeppelin models similar, as we might expect for coherently oriented fibers in the CC, causing the BIC generally to prefer the simpler Zeppelin models.

Cylinder models in group (i) consistently provide axon diameter index values of around 5 μm, which is consistent with axon diameter estimates from the CC in 6,9. Other models show more erratic estimates of radius which arise because the models fit the data less well, and so use the parameter to explain effects they do not capture. The GDR-cylinders models' shape parameter *κ* often hits the upper bound constrained in the fitting to 10. At this value of *κ*, the Gamma distribution is close to Gaussian shape and is highly peaked about the mean, making the GDR-cylinders very similar to the Cylinder model. BIC, thus, prefers the simpler Cylinder model. The Sphere and Astrocylinder radius estimate is usually around 0.1 μm, which makes them very similar to the simpler Dot and Astrosticks models, respectively, which the BIC generally prefers.

We also compared (see Supporting Information) model parameter estimates from the different data sets, 2 × 4 h and 8 × 1 h, and different *η*. The CC voxels selected for averaging were different in each data set (see subsection “Voxel Selection”), producing some variation. In particular, the axon radius index was higher in the 8 × 1 h data set, which we expect because it has a greater contribution from the midbody where axons are larger. However, the estimates obtained from multiple sessions (8 × 1 h data) were broadly in line with those of the two-session data (2 × 4 h data).

As *η* increased, the LSE went down because the number of voxels being averaged increased, which increases the SNR. We saw a slight increase in the radius estimate and decrease in axial diffusivity as dispersion increased, but the effects were minor.

## DISCUSSION

This note reports a similar experiment to Panagiotaki et al. 11 but using in vivo human data rather than fixed rat tissue. We also extended the analysis to determine ranking stability with respect to noise, protocol, and model selection technique. We sampled a wide range of *b*-values and diffusion times achievable on a clinical system and also used a much higher angular resolution sampling than 11. The overall ranking we obtain is similar to previous observations from fixed tissue 11, with a few differences. Though there are minor differences due to intersessional variability and subsequent image registration, the similarity between 2 × 4 h and 8 × 1 h data sets is important because it means we can construct data sets for this kind of experiment from multiple short sessions, which are much more comfortable for the participant. The additional steps in the analysis reveal a group structure to the model ranking and suggest that the models in group (i) perform similarly well in explaining the full range of Pulsed-Gradient Spin-Echo signals acquirable from the human brain on current clinical systems. The finding supports methods like 6–8 which estimate axon density and diameter using these kinds of models.

The experiment here uses only data from the CC, which is relatively homogeneous, with little fiber dispersion, crossing or cerebrospinal fluid contamination. However, these effects may still influence the measurement to some extent. Moreover, the greater angular threshold increases fiber dispersion, which is reflected in the fitting and parameter estimates, and which none of the models we test here are designed to capture. The intention here is to start with the simplest geometry before performing a similar analysis in more complex regions. Even in the CC, more sophisticated models may outperform the limited set we study here; models that explicitly cater for fiber features such as dispersion/crossing 10,19,22, cerebrospinal fluid pool as in 7,10, or permeability 3, will be the focus of further work, as will the exploration of other white matter regions.

Another limitation of this study is that the results presented in this article were obtained from just one subject. It is important to note, therefore, that we are unable to say whether the same results would be obtained in another participant, or from another scanning session. Future work will also explore improvements to the fitting procedure, for example, to account better for the varying noise levels across shells with different echo time, although we expect these to have only minor effects on the results.

The protocol we use here is designed specifically for model selection rather than large scale application. It is true that reduced data sets will favor simpler models and possibly larger data sets will support even more complex models. Here we sampled as widely as possible the measurement space to get the best idea of which model explains better the entire measurement space. Once appropriate models have been identified, experiment design techniques such as 23 can determine economical protocols for widespread use. We emphasize that the choice of models our analysis suggests is not appropriate for existing sparse data sets such as off-the-shelf single shell high angular resolution diffusion imaging data, which only support simple models. Rather, these results inform the choice of protocol for future in vivo microstructure imaging once we identify the right model.
