# Supplementary material for: A ranking of diffusion MRI compartment models with in vivo human brain data
Source: Magn Reson Med. 2013 Dec 17;72(6):1785–92. doi: 10.1002/mrm.25080 (PMC4278549; doi:10.1002/mrm.25080)
Supplement: Supplementary file 1 — Supplementary Information [file mrm0072-1785-SD1.docx]

| ***LSE*** | ***S_0_*** | ***Nr Parameters*** |  | ***Vol. Fraction*** | ***κ (Gamma Dist.)*** | ***Diameter / x10^-6^ m*** | ***Vol. Fraction*** | ***Axial Diff. / x10^-9^ m^2^/s*** | ***Radial Diff.1 / x10^-9^ m^2^/s*** | ***Radial Diff.2 / x10^-9^ m^2^/s*** | ***Theta/ arc degree°*** | ***Phi/ arc degree°*** | ***Alpha/ arc degree°*** | ***Vol. Fraction*** | ***Diameter / x10^-6^ m*** |
| --- | --- | --- | --- | --- | --- | --- | --- | --- | --- | --- | --- | --- | --- | --- | --- |
|  |  |  | ***Models*** | ***Stick/Cylind.*** | | | ***Tensor/Zeppelin/Ball*** | | | | | | | ***3rd Compart.*** | |
| 761 | 0.96 | 7 | **ZeppelinStickDot** | 0.29 |  |  | 0.62 | 1.91 | 0.68 |  | 88.9 | 0.8 |  | 0.09 |  |
| 748 | 0.96 | 9 | **TensorStickDot** | 0.29 |  |  | 0.62 | 1.91 | 0.73 | 0.63 | 88.9 | 0.8 | 10.8 | 0.09 |  |
| 761 | 0.96 | 8 | **ZeppelinCylinderDot** | 0.30 |  | 4.45 | 0.62 | 1.91 | 0.68 |  | 88.8 | 0.9 |  | 0.09 |  |
| 761 | 0.96 | 8 | **ZeppelinStickSphere** | 0.29 |  |  | 0.62 | 1.91 | 0.68 |  | 88.8 | 0.7 |  | 0.09 | 0.20 |
| 748 | 0.96 | 10 | **TensorCylinderDot** | 0.29 |  | 4.36 | 0.62 | 1.91 | 0.73 | 0.63 | 88.9 | 0.8 | 10.8 | 0.09 |  |
| 748 | 0.96 | 10 | **TensorStickSphere** | 0.29 |  |  | 0.62 | 1.91 | 0.73 | 0.63 | 88.9 | 0.8 | 10.8 | 0.09 | 0.21 |
| 761 | 0.96 | 9 | **ZeppelinCylinderSphere** | 0.30 |  | 4.45 | 0.62 | 1.91 | 0.68 |  | 88.9 | 0.8 |  | 0.09 | 0.20 |
| 761 | 0.96 | 9 | **ZeppelinGDRCylindersDot** | 0.30 | 7.0 | 4.19 | 0.62 | 1.91 | 0.68 |  | 88.8 | 0.5 |  | 0.09 |  |
| 748 | 0.96 | 11 | **TensorCylinderSphere** | 0.29 |  | 4.34 | 0.62 | 1.91 | 0.73 | 0.63 | 88.9 | 0.8 | 11.3 | 0.09 | 0.10 |
| 748 | 0.96 | 11 | **TensorGDRCylindersDot** | 0.29 | 10.0 | 4.14 | 0.62 | 1.91 | 0.73 | 0.63 | 88.9 | 0.8 | 10.8 | 0.09 |  |
| 761 | 0.96 | 10 | **ZeppelinGDRCylindersSphere** | 0.30 | 10.0 | 4.26 | 0.62 | 1.91 | 0.68 |  | 88.8 | 0.8 |  | 0.09 | 0.20 |
| 748 | 0.96 | 12 | **TensorGDRCylindersSphere** | 0.29 | 10.0 | 4.14 | 0.62 | 1.91 | 0.73 | 0.63 | 88.9 | 0.8 | 10.8 | 0.09 | 0.20 |
| 909 | 0.97 | 7 | **ZeppelinStickAstrosticks** | 0.33 |  |  | 0.42 | 2.06 | 0.67 |  | 88.8 | 0.6 |  | 0.25 |  |
| 895 | 0.96 | 9 | **TensorStickAstrosticks** | 0.33 |  |  | 0.43 | 2.06 | 0.73 | 0.58 | 88.9 | 0.7 | 11.2 | 0.25 |  |
| 909 | 0.97 | 8 | **ZeppelinCylinderAstrosticks** | 0.33 |  | 3.96 | 0.42 | 2.06 | 0.67 |  | 88.9 | 0.7 |  | 0.25 |  |
| 909 | 0.97 | 8 | **ZeppelinStickAstrocylinders** | 0.33 |  |  | 0.42 | 2.06 | 0.67 |  | 88.8 | 0.6 |  | 0.25 | 0.20 |
| 909 | 0.97 | 8 | **ZeppelinCylinderAstrocylinders** | 0.33 |  | 0.20 | 0.42 | 2.06 | 0.67 |  | 88.9 | 0.7 |  | 0.25 | 0.20 |
| 895 | 0.96 | 10 | **TensorCylinderAstrosticks** | 0.33 |  | 3.78 | 0.42 | 2.06 | 0.73 | 0.58 | 88.9 | 0.7 | 11.2 | 0.25 |  |
| 895 | 0.96 | 10 | **TensorStickAstrocylinders** | 0.33 |  |  | 0.43 | 2.06 | 0.73 | 0.58 | 88.9 | 0.7 | 11.3 | 0.25 | 0.25 |
| 895 | 0.96 | 10 | **TensorCylinderAstrocylinders** | 0.33 |  | 0.25 | 0.43 | 2.06 | 0.73 | 0.58 | 88.9 | 0.7 | 11.2 | 0.25 | 0.25 |
| 909 | 0.97 | 9 | **ZeppelinGDRCylindersAstrosticks** | 0.33 | 5.2 | 3.82 | 0.42 | 2.06 | 0.67 |  | 88.9 | 0.7 |  | 0.25 |  |
| 909 | 0.97 | 9 | **ZeppelinGDRCylindersAstrocylinders** | 0.33 | 1.4 | 0.63 | 0.42 | 2.06 | 0.67 |  | 88.9 | 0.9 |  | 0.25 | 0.63 |
| 895 | 0.96 | 11 | **TensorGDRCylindersAstrosticks** | 0.33 | 10.0 | 3.60 | 0.42 | 2.06 | 0.73 | 0.58 | 88.9 | 0.7 | 11.2 | 0.25 |  |
| 895 | 0.96 | 11 | **TensorGDRCylindersAstrocylinders** | 0.33 | 1.8 | 0.20 | 0.43 | 2.06 | 0.73 | 0.58 | 88.9 | 0.7 | 11.2 | 0.25 | 0.20 |
| 1028 | 0.97 | 7 | **Bizeppelin** | 0.63 |  |  | 0.37 | 1.47 | 1.47 |  | 88.8 | 0.9 |  |  |  |
| 1076 | 0.99 | 8 | **BallGDRCylindersDot** | 0.66 | 1.1 | 19.42 | 0.27 | 1.77 |  |  | 88.8 | 0.7 |  | 0.07 |  |
| 1080 | 0.99 | 8 | **BallGDRCylindersAstrosticks** | 0.56 | 1.1 | 16.33 | 0.20 | 1.99 |  |  | 88.9 | 0.7 |  | 0.24 |  |
| 1076 | 0.99 | 9 | **BallGDRCylindersSphere** | 0.66 | 1.1 | 19.42 | 0.27 | 1.77 |  |  | 88.8 | 0.7 |  | 0.07 | 1.00 |
| 1099 | 0.99 | 7 | **BallCylinderAstrosticks** | 0.53 |  | 12.60 | 0.22 | 2.01 |  |  | 88.9 | 0.6 |  | 0.24 |  |
| 1133 | 0.95 | 6 | **ZeppelinStick** | 0.40 |  |  | 0.60 | 1.49 | 0.72 |  | 88.9 | 0.8 |  |  |  |
| 1120 | 0.95 | 8 | **TensorStick** | 0.40 |  |  | 0.60 | 1.49 | 0.76 | 0.66 | 88.9 | 0.8 | 9.6 |  |  |
| 1128 | 1.00 | 7 | **BallCylinderDot** | 0.60 |  | 13.42 | 0.33 | 1.75 |  |  | 88.8 | 0.7 |  | 0.07 |  |
| 1132 | 0.95 | 7 | **ZeppelinCylinder** | 0.41 |  | 4.83 | 0.59 | 1.49 | 0.72 |  | 88.9 | 0.8 |  |  |  |
| 1120 | 0.95 | 9 | **TensorCylinder** | 0.41 |  | 4.71 | 0.59 | 1.49 | 0.76 | 0.66 | 88.9 | 0.8 | 9.6 |  |  |
| 1128 | 1.00 | 8 | **BallCylinderSphere** | 0.60 |  | 13.42 | 0.33 | 1.75 |  |  | 88.8 | 0.7 |  | 0.07 | 0.24 |
| 1132 | 0.95 | 8 | **ZeppelinGDRCylinders** | 0.41 | 10.0 | 4.67 | 0.59 | 1.49 | 0.72 |  | 88.9 | 0.8 |  |  |  |
| 1120 | 0.95 | 10 | **TensorGDRCylinders** | 0.41 | 10.0 | 4.57 | 0.59 | 1.49 | 0.76 | 0.66 | 88.9 | 0.8 | 9.7 |  |  |
| 1180 | 0.99 | 7 | **BallCylinderAstrocylinders** | 0.50 |  | 11.04 | 0.24 | 1.95 |  |  | 88.9 | 0.6 |  | 0.27 | 11.04 |
| 1181 | 0.99 | 8 | **BallGDRCylindersAstrocylinders** | 0.50 | 10.0 | 11.12 | 0.24 | 1.95 |  |  | 88.9 | 0.6 |  | 0.27 | 11.12 |
| 1311 | 0.98 | 7 | **BallGDRCylinders** | 0.65 | 1.1 | 14.82 | 0.35 | 1.44 |  |  | 88.7 | 0.8 |  |  |  |
| 1335 | 1.00 | 7 | **BallStickAstrocylinders** | 0.41 |  |  | 0.31 | 1.93 |  |  | 89.0 | 0.6 |  | 0.28 | 10.38 |
| 1344 | 1.00 | 6 | **BallStickAstrosticks** | 0.41 |  |  | 0.36 | 1.86 |  |  | 89.0 | 0.7 |  | 0.23 |  |
| 1345 | 0.98 | 6 | **BallCylinder** | 0.61 |  | 11.26 | 0.39 | 1.43 |  |  | 88.8 | 0.7 |  |  |  |
| 1455 | 1.00 | 7 | **BallStickSphere** | 0.43 |  |  | 0.46 | 1.61 |  |  | 88.9 | 0.7 |  | 0.10 | 17.90 |
| 1492 | 0.99 | 6 | **BallStickDot** | 0.44 |  |  | 0.52 | 1.49 |  |  | 88.9 | 0.8 |  | 0.04 |  |
| 1548 | 0.98 | 5 | **BallStick** | 0.48 |  |  | 0.52 | 1.35 |  |  | 88.9 | 0.8 |  |  |  |
| 2071 | 0.89 | 7 | **DT** |  |  |  | 0.85 | 1.54 | 0.19 | 0.23 | 88.8 | 0.9 | 13.9 |  |  |

**Supplementary Table 1:** Parameter estimates obtained after fitting models to the 2x4h 2^o^ data set. The models are ordered top-down by the *BIC* score. For GDR-Cylinder models, we report the mean of the radius distribution; the number of model parameters includes the parameter S_0_, which is the unweighted signal at b=0; we report the Fractional Anisotropy under DT's volume fraction column. Angles Theta/Phi/Alpha give the spatial orientation.
