# Supplementary material for: A ranking of diffusion MRI compartment models with in vivo human brain data
Source: Magn Reson Med. 2013 Dec 17;72(6):1785–92. doi: 10.1002/mrm.25080 (PMC4278549; doi:10.1002/mrm.25080)
Supplement: Supplementary file 2 — Supplementary Information [file mrm0072-1785-SD2.docx]

|  | ***2x4_2*** | ***2x4_5*** | ***2x4_10*** | ***8x1_2*** | ***8x1_5*** | ***8x1_10*** | ***2x4_2*** | ***2x4_5*** | ***2x4_10*** | ***8x1_2*** | ***8x1_5*** | ***8x1_10*** | ***2x4_2*** | ***2x4_5*** | ***2x4_10*** | ***8x1_2*** | ***8x1_5*** | ***8x1_10*** | ***2x4_2*** | ***2x4_5*** | ***2x4_10*** | ***8x1_2*** | ***8x1_5*** | ***8x1_10*** | ***2x4_2*** | ***2x4_5*** | ***2x4_10*** | ***8x1_2*** | ***8x1_5*** | ***8x1_10*** |
| --- | --- | --- | --- | --- | --- | --- | --- | --- | --- | --- | --- | --- | --- | --- | --- | --- | --- | --- | --- | --- | --- | --- | --- | --- | --- | --- | --- | --- | --- | --- |
| ***Models*** | ***BIC*** | | | | | | ***Stick/Cyl. Volume Fraction*** | | | | | | ***Axial Diff. (x10^-9^m^2^/s)*** | | | | | | ***Radial Diff. (x10^-9^m^2^/s)*** | | | | | | ***Cylinder Diameter (x10^-6^m)*** | | | | | |
| **ZeppelinStickDot** | 813 | 626 | 599 | 877 | 881 | 802 | 0.29 | 0.30 | 0.29 | 0.30 | 0.29 | 0.27 | 1.91 | 1.85 | 1.82 | 1.81 | 1.77 | 1.75 | 0.68 | 0.67 | 0.66 | 0.64 | 0.62 | 0.64 |  |  |  |  |  |  |
| **TensorStickDot** | 814 | 628 | 605 | 853 | 859 | 796 | 0.29 | 0.29 | 0.29 | 0.29 | 0.28 | 0.27 | 1.91 | 1.85 | 1.82 | 1.81 | 1.78 | 1.75 | 0.73 | 0.71 | 0.69 | 0.71 | 0.68 | 0.69 |  |  |  |  |  |  |
| **ZeppelinCylinderDot** | 820 | 633 | 605 | 875 | 856 | 788 | 0.30 | 0.30 | 0.30 | 0.35 | 0.38 | 0.35 | 1.91 | 1.85 | 1.82 | 1.81 | 1.77 | 1.75 | 0.68 | 0.67 | 0.66 | 0.66 | 0.67 | 0.68 | 4.4 | 5.4 | 6.9 | 9.3 | 11.0 | 10.5 |
| **ZeppelinStickSphere** | 820 | 634 | 606 | 884 | 883 | 808 | 0.29 | 0.30 | 0.29 | 0.30 | 0.29 | 0.28 | 1.91 | 1.85 | 1.82 | 1.81 | 1.80 | 1.77 | 0.68 | 0.67 | 0.66 | 0.64 | 0.63 | 0.64 |  |  |  |  |  |  |
| **TensorCylinderDot** | 821 | 635 | 611 | 852 | 836 | 783 | 0.29 | 0.30 | 0.30 | 0.34 | 0.37 | 0.35 | 1.91 | 1.85 | 1.82 | 1.81 | 1.78 | 1.76 | 0.73 | 0.71 | 0.70 | 0.73 | 0.74 | 0.73 | 4.4 | 5.3 | 6.8 | 9.2 | 10.9 | 10.4 |
| **TensorStickSphere** | 821 | 635 | 612 | 860 | 861 | 802 | 0.29 | 0.29 | 0.29 | 0.29 | 0.28 | 0.27 | 1.91 | 1.85 | 1.82 | 1.81 | 1.81 | 1.77 | 0.73 | 0.71 | 0.69 | 0.71 | 0.69 | 0.69 |  |  |  |  |  |  |
| **ZeppelinCylinderSphere** | 827 | 641 | 612 | 882 | 863 | 796 | 0.30 | 0.30 | 0.30 | 0.35 | 0.38 | 0.35 | 1.91 | 1.85 | 1.82 | 1.81 | 1.77 | 1.75 | 0.68 | 0.67 | 0.66 | 0.66 | 0.67 | 0.68 | 4.4 | 5.4 | 6.9 | 9.3 | 11.0 | 10.5 |
| **ZeppelinGDRCylindersDot** | 827 | 641 | 613 | 882 | 863 | 796 | 0.30 | 0.30 | 0.30 | 0.35 | 0.38 | 0.35 | 1.91 | 1.85 | 1.82 | 1.81 | 1.77 | 1.75 | 0.68 | 0.67 | 0.66 | 0.66 | 0.67 | 0.68 | 4.2 | 5.2 | 6.8 | 9.4 | 11.2 | 10.7 |
| **TensorCylinderSphere** | 828 | 642 | 618 | 859 | 843 | 791 | 0.29 | 0.30 | 0.30 | 0.34 | 0.37 | 0.35 | 1.91 | 1.85 | 1.82 | 1.81 | 1.78 | 1.76 | 0.73 | 0.71 | 0.70 | 0.73 | 0.74 | 0.73 | 4.3 | 5.3 | 6.8 | 9.2 | 10.9 | 10.4 |
| **TensorGDRCylindersDot** | 828 | 642 | 618 | 859 | 843 | 791 | 0.29 | 0.30 | 0.30 | 0.34 | 0.38 | 0.35 | 1.91 | 1.85 | 1.82 | 1.81 | 1.78 | 1.76 | 0.73 | 0.71 | 0.70 | 0.73 | 0.74 | 0.73 | 4.1 | 5.1 | 6.7 | 9.2 | 11.1 | 10.6 |
| **ZeppelinGDRCylindersSphere** | 835 | 648 | 620 | 889 | 870 | 803 | 0.30 | 0.30 | 0.30 | 0.35 | 0.38 | 0.35 | 1.91 | 1.85 | 1.82 | 1.81 | 1.77 | 1.75 | 0.68 | 0.67 | 0.66 | 0.66 | 0.67 | 0.68 | 4.3 | 5.2 | 6.8 | 9.4 | 11.2 | 10.7 |
| **TensorGDRCylindersSphere** | 836 | 650 | 626 | 867 | 851 | 798 | 0.29 | 0.30 | 0.30 | 0.34 | 0.38 | 0.35 | 1.91 | 1.85 | 1.82 | 1.81 | 1.78 | 1.76 | 0.73 | 0.71 | 0.70 | 0.73 | 0.74 | 0.73 | 4.1 | 5.1 | 6.7 | 9.2 | 11.1 | 10.6 |
| **ZeppelinStickAstrosticks** | 961 | 792 | 767 | 1046 | 1053 | 987 | 0.33 | 0.33 | 0.33 | 0.34 | 0.32 | 0.31 | 2.06 | 1.97 | 1.94 | 1.90 | 1.89 | 1.87 | 0.67 | 0.66 | 0.65 | 0.63 | 0.61 | 0.64 |  |  |  |  |  |  |
| **TensorStickAstrosticks** | 961 | 793 | 772 | 1021 | 1029 | 981 | 0.33 | 0.33 | 0.32 | 0.33 | 0.32 | 0.31 | 2.06 | 1.98 | 1.94 | 1.91 | 1.91 | 1.88 | 0.73 | 0.72 | 0.70 | 0.72 | 0.69 | 0.71 |  |  |  |  |  |  |
| **ZeppelinCylinderAstrosticks** | 968 | 799 | 773 | 1045 | 1029 | 975 | 0.33 | 0.34 | 0.34 | 0.39 | 0.42 | 0.39 | 2.06 | 1.97 | 1.94 | 1.89 | 1.88 | 1.87 | 0.67 | 0.67 | 0.66 | 0.66 | 0.68 | 0.69 | 4.0 | 5.0 | 6.7 | 9.1 | 10.7 | 10.2 |
| **ZeppelinStickAstrocyl.** | 968 | 800 | 775 | 1054 | 1060 | 995 | 0.33 | 0.33 | 0.33 | 0.34 | 0.32 | 0.31 | 2.06 | 1.97 | 1.94 | 1.90 | 1.89 | 1.87 | 0.67 | 0.66 | 0.65 | 0.63 | 0.61 | 0.64 |  |  |  |  |  |  |
| **ZeppelinCylinderAstrocyl.** | 968 | 800 | 775 | 1054 | 1052 | 992 | 0.33 | 0.33 | 0.33 | 0.34 | 0.36 | 0.33 | 2.06 | 1.97 | 1.94 | 1.89 | 1.89 | 1.87 | 0.67 | 0.67 | 0.65 | 0.63 | 0.61 | 0.64 | 0.2 | 0.2 | 0.5 | 4.5 | 8.1 | 6.9 |
| **TensorCylinderAstrosticks** | 968 | 800 | 779 | 1021 | 1009 | 970 | 0.33 | 0.33 | 0.34 | 0.37 | 0.41 | 0.38 | 2.06 | 1.98 | 1.94 | 1.91 | 1.90 | 1.87 | 0.73 | 0.73 | 0.71 | 0.75 | 0.77 | 0.77 | 3.8 | 4.9 | 6.6 | 8.9 | 10.6 | 10.1 |
| **TensorStickAstrocyl.** | 969 | 801 | 780 | 1029 | 1037 | 988 | 0.33 | 0.33 | 0.32 | 0.33 | 0.32 | 0.31 | 2.06 | 1.98 | 1.94 | 1.91 | 1.91 | 1.88 | 0.73 | 0.72 | 0.70 | 0.72 | 0.69 | 0.71 |  |  |  |  |  |  |
| **TensorCylinderAstrocyl.** | 969 | 801 | 780 | 1029 | 1029 | 986 | 0.33 | 0.33 | 0.32 | 0.33 | 0.35 | 0.33 | 2.06 | 1.98 | 1.94 | 1.91 | 1.90 | 1.88 | 0.73 | 0.72 | 0.70 | 0.72 | 0.70 | 0.71 | 0.3 | 0.2 | 0.4 | 4.4 | 8.0 | 6.9 |
| **ZeppelinGDRCylindersAstrost.** | 975 | 807 | 781 | 1052 | 1037 | 982 | 0.33 | 0.34 | 0.34 | 0.39 | 0.42 | 0.39 | 2.06 | 1.97 | 1.94 | 1.89 | 1.88 | 1.87 | 0.67 | 0.67 | 0.66 | 0.66 | 0.68 | 0.69 | 3.8 | 5.1 | 6.6 | 9.1 | 10.9 | 10.3 |
| **ZeppelinGDRCylindersAstrocyl.** | 975 | 807 | 782 | 1061 | 1060 | 999 | 0.33 | 0.34 | 0.33 | 0.34 | 0.36 | 0.33 | 2.06 | 1.97 | 1.94 | 1.89 | 1.89 | 1.87 | 0.67 | 0.66 | 0.66 | 0.63 | 0.61 | 0.64 | 0.6 | 0.2 | 0.2 | 4.5 | 8.0 | 6.9 |
| **TensorGDRCylindersAstrost.** | 976 | 808 | 786 | 1029 | 1016 | 978 | 0.33 | 0.33 | 0.34 | 0.37 | 0.41 | 0.38 | 2.06 | 1.98 | 1.94 | 1.91 | 1.90 | 1.87 | 0.73 | 0.73 | 0.71 | 0.75 | 0.77 | 0.77 | 3.6 | 4.7 | 6.5 | 8.9 | 10.8 | 10.2 |
| **TensorGDRCylindersAstrocyl.** | 976 | 808 | 787 | 1036 | 1037 | 993 | 0.33 | 0.33 | 0.32 | 0.33 | 0.35 | 0.33 | 2.06 | 1.98 | 1.94 | 1.91 | 1.90 | 1.88 | 0.73 | 0.72 | 0.70 | 0.72 | 0.70 | 0.71 | 0.2 | 0.2 | 0.5 | 4.5 | 8.0 | 6.8 |
| **Bizeppelin** | 1079 | 954 | 941 | 1222 | 1265 | 1223 | 0.63 | 0.60 | 0.59 | 0.54 | 0.54 | 0.54 | 1.47 | 1.42 | 1.39 | 1.41 | 1.35 | 1.32 | 1.47 | 1.22 | 1.15 | 0.04 | 0.05 | 0.05 |  |  |  |  |  |  |
| **BallGDRCylindersDot** | 1135 | 940 | 894 | 1162 | 1063 | 1002 | 0.66 | 0.67 | 0.68 | 0.71 | 0.72 | 0.70 | 1.77 | 1.72 | 1.68 | 1.66 | 1.65 | 1.63 |  |  |  |  |  |  | 19.4 | 19.9 | 20.2 | 20.2 | 20.2 | 20.2 |
| **BallGDRCylindersAstrosticks** | 1139 | 965 | 929 | 1206 | 1124 | 1072 | 0.56 | 0.57 | 0.58 | 0.64 | 0.64 | 0.62 | 1.99 | 1.92 | 1.88 | 1.86 | 1.85 | 1.83 |  |  |  |  |  |  | 16.3 | 16.6 | 17.2 | 18.4 | 19.1 | 19.1 |
| **BallGDRCylindersSphere** | 1142 | 947 | 902 | 1169 | 1071 | 1010 | 0.66 | 0.67 | 0.68 | 0.71 | 0.72 | 0.70 | 1.77 | 1.71 | 1.68 | 1.66 | 1.65 | 1.63 |  |  |  |  |  |  | 19.4 | 19.8 | 20.2 | 20.2 | 20.2 | 20.2 |
| **BallCylinderAstrosticks** | 1151 | 983 | 948 | 1232 | 1143 | 1101 | 0.53 | 0.54 | 0.54 | 0.58 | 0.59 | 0.56 | 2.01 | 1.93 | 1.89 | 1.86 | 1.85 | 1.83 |  |  |  |  |  |  | 12.6 | 12.6 | 12.8 | 13.2 | 13.5 | 13.4 |
| **ZeppelinStick** | 1177 | 1016 | 997 | 1249 | 1305 | 1254 | 0.40 | 0.41 | 0.40 | 0.41 | 0.40 | 0.40 | 1.49 | 1.43 | 1.40 | 1.41 | 1.36 | 1.33 | 0.72 | 0.70 | 0.69 | 0.67 | 0.65 | 0.67 |  |  |  |  |  |  |
| **TensorStick** | 1179 | 1019 | 1004 | 1229 | 1286 | 1250 | 0.40 | 0.41 | 0.40 | 0.41 | 0.40 | 0.40 | 1.49 | 1.43 | 1.40 | 1.41 | 1.36 | 1.33 | 0.76 | 0.75 | 0.73 | 0.74 | 0.72 | 0.73 |  |  |  |  |  |  |
| **BallCylinderDot** | 1179 | 995 | 951 | 1222 | 1113 | 1063 | 0.60 | 0.60 | 0.61 | 0.64 | 0.65 | 0.63 | 1.75 | 1.69 | 1.66 | 1.65 | 1.64 | 1.62 |  |  |  |  |  |  | 13.4 | 13.4 | 13.6 | 13.9 | 14.3 | 14.3 |
| **ZeppelinCylinder** | 1184 | 1022 | 1001 | 1242 | 1269 | 1232 | 0.41 | 0.42 | 0.43 | 0.47 | 0.51 | 0.48 | 1.49 | 1.43 | 1.40 | 1.41 | 1.36 | 1.33 | 0.72 | 0.71 | 0.71 | 0.71 | 0.73 | 0.73 | 4.8 | 5.6 | 6.7 | 8.5 | 9.8 | 9.3 |
| **TensorCylinder** | 1186 | 1025 | 1008 | 1223 | 1253 | 1229 | 0.41 | 0.42 | 0.43 | 0.46 | 0.50 | 0.48 | 1.49 | 1.43 | 1.40 | 1.41 | 1.36 | 1.33 | 0.76 | 0.75 | 0.74 | 0.78 | 0.80 | 0.79 | 4.7 | 5.5 | 6.6 | 8.4 | 9.7 | 9.2 |
| **BallCylinderSphere** | 1186 | 1002 | 958 | 1229 | 1120 | 1070 | 0.60 | 0.60 | 0.61 | 0.64 | 0.65 | 0.63 | 1.75 | 1.69 | 1.66 | 1.65 | 1.64 | 1.62 |  |  |  |  |  |  | 13.4 | 13.4 | 13.6 | 13.9 | 14.3 | 14.3 |
| **ZeppelinGDRCylinders** | 1191 | 1029 | 1008 | 1249 | 1276 | 1239 | 0.41 | 0.42 | 0.43 | 0.47 | 0.51 | 0.49 | 1.49 | 1.43 | 1.40 | 1.41 | 1.36 | 1.33 | 0.72 | 0.71 | 0.71 | 0.71 | 0.73 | 0.74 | 4.7 | 5.5 | 6.7 | 8.6 | 10.0 | 9.4 |
| **TensorGDRCylinders** | 1193 | 1033 | 1016 | 1230 | 1261 | 1237 | 0.41 | 0.42 | 0.43 | 0.46 | 0.50 | 0.48 | 1.49 | 1.43 | 1.40 | 1.41 | 1.36 | 1.33 | 0.76 | 0.75 | 0.74 | 0.78 | 0.80 | 0.79 | 4.6 | 5.4 | 6.6 | 8.4 | 9.8 | 9.4 |
| **BallCylinderAstrocylinders** | 1231 | 1064 | 1036 | 1319 | 1239 | 1197 | 0.50 | 0.50 | 0.51 | 0.55 | 0.54 | 0.52 | 1.95 | 1.88 | 1.83 | 1.79 | 1.80 | 1.77 |  |  |  |  |  |  | 11.0 | 11.0 | 11.2 | 11.8 | 12.0 | 11.8 |
| **BallGDRCylindersAstrocyl.** | 1240 | 1073 | 1045 | 1328 | 1249 | 1206 | 0.50 | 0.50 | 0.51 | 0.55 | 0.54 | 0.52 | 1.95 | 1.87 | 1.83 | 1.78 | 1.80 | 1.77 |  |  |  |  |  |  | 11.1 | 11.1 | 11.3 | 12.0 | 12.1 | 11.9 |
| **BallGDRCylinders** | 1362 | 1190 | 1153 | 1396 | 1357 | 1319 | 0.65 | 0.66 | 0.67 | 0.71 | 0.73 | 0.70 | 1.44 | 1.38 | 1.36 | 1.37 | 1.33 | 1.30 |  |  |  |  |  |  | 14.8 | 14.8 | 15.3 | 16.3 | 17.0 | 16.7 |
| **BallStickAstrocylinders** | 1387 | 1225 | 1217 | 1568 | 1504 | 1429 | 0.41 | 0.41 | 0.41 | 0.42 | 0.41 | 0.40 | 1.93 | 1.85 | 1.81 | 1.80 | 1.85 | 1.79 |  |  |  |  |  |  |  |  |  |  |  |  |
| **BallStickAstrosticks** | 1388 | 1228 | 1227 | 1613 | 1580 | 1483 | 0.41 | 0.41 | 0.41 | 0.43 | 0.41 | 0.40 | 1.86 | 1.78 | 1.72 | 1.64 | 1.66 | 1.62 |  |  |  |  |  |  |  |  |  |  |  |  |
| **BallCylinder** | 1389 | 1220 | 1184 | 1428 | 1386 | 1353 | 0.61 | 0.62 | 0.62 | 0.66 | 0.67 | 0.65 | 1.43 | 1.38 | 1.35 | 1.36 | 1.32 | 1.29 |  |  |  |  |  |  | 11.3 | 11.2 | 11.3 | 11.8 | 12.1 | 11.9 |
| **BallStickSphere** | 1507 | 1330 | 1312 | 1638 | 1567 | 1489 | 0.43 | 0.43 | 0.43 | 0.44 | 0.43 | 0.41 | 1.61 | 1.55 | 1.53 | 1.54 | 1.58 | 1.53 |  |  |  |  |  |  |  |  |  |  |  |  |
| **BallStickDot** | 1536 | 1360 | 1351 | 1719 | 1710 | 1594 | 0.44 | 0.44 | 0.43 | 0.45 | 0.44 | 0.43 | 1.49 | 1.44 | 1.40 | 1.36 | 1.34 | 1.33 |  |  |  |  |  |  |  |  |  |  |  |  |
| **BallStick** | 1584 | 1415 | 1404 | 1743 | 1761 | 1662 | 0.48 | 0.48 | 0.47 | 0.48 | 0.48 | 0.47 | 1.35 | 1.29 | 1.26 | 1.26 | 1.22 | 1.19 |  |  |  |  |  |  |  |  |  |  |  |  |
| **DT** | 2123 | 2001 | 1984 | 2208 | 2206 | 2247 | 0.85 | 0.84 | 0.84 | 0.85 | 0.84 | 0.83 | 1.54 | 1.48 | 1.45 | 1.46 | 1.40 | 1.38 | 0.19 | 0.19 | 0.19 | 0.17 | 0.18 | 0.19 |  |  |  |  |  |  |

**Supplementary Table 2:** Various model parameters from different data sets, 2x4h and 8x1h, with different angular thresholds of 2^o^, 5^o^, and 10^o^ . The notation is as in Table 1. Regarding the *BIC*, Raftery [19] regard the preference for one model against another as `weak' when their *BIC* difference is 0-2, `positive' for 2-6, `strong' for 6-10, and as `very strong' for anything above 10.
